# Abundance and characteristics of anthropogenic litter along the Tanzanian shores of the African Great Lakes: including volunteer involvement, outreach, and stakeholder engagement in a holistic approach

**DOI:** 10.1007/s11356-025-36528-8

**Published:** 2025-05-20

**Authors:** Bahati S. Mayoma, Mamlo A. Yusuph, Hellena Sailas, Zagalo Emanuel, Editrudith Lukanga, Lawrence Kitogo, Adventina A. Edson, Deogratius Simbila, Arthur Mugema, Paul Matonya, Fanuel Kasenene, Christina Sørensen, Conrad Sparks, Farhan R. Khan

**Affiliations:** 1 School of Aquatic Sciences and Fisheries Technology, University of Dar Es Salaam, P.O Box 60091, Dar Es Salaam, Tanzania; 2 Environmental Conservation Community of Tanzania, Mbezi Beach, P.O Box 70132, Dar Es Salaam, Tanzania; 3 Arena Recycling Industry, P.O Box 17108, Dar Es Salaam, Tanzania; 4 Environmental Management and Economic Development Organization (EMEDO), P.O Box 2964, Mwanza, Tanzania; 5 Sustainable Ocean Alliance (SOA) Tanzania, P.O Box 3032, Dar Es Salaam, Tanzania; 6 Natural Resource and Environment Conservation Section, Mwanza City Council, P.O Box 1333, Mwanza, Tanzania; 7 Norwegian Institute for Water Research (NIVA), Thormøhlensgate 53D, NO-5006, Bergen, Norway; 8 Centre for Sustainable Oceans, Faculty of Applied Sciences, Cape Peninsula University of Technology District Six Campus, Cape Town, South Africa; 9 Department of Climate & Environment, NORCE Norwegian Research Centre AS, Nygårdsporten 112, NO-5008, Bergen, Norway

**Keywords:** Anthropogenic litter, Plastic pollution, Citizen science, Outreach and education, Freshwater, East Africa

## Abstract

Environmental pollution due to mismanaged anthropogenic litter is one of the most pressing issues facing the world today. To reduce existing environmental burdens of litter, clean-up events have increased in popularity. Here, we report on the results of the Clean Shores, Great Lakes project, in which we conducted 69 volunteer-based clean-ups along the Tanzanian shorelines of the African Great Lakes (Victoria, Tanganyika, and Nyasa (also known as Lake Malawi)). A total of 5483 volunteers engaged with the project across all clean-up sites, collecting 431,328 pieces (25,981 kg) of anthropogenic litter. Expressing the data as normalized to area (items m^−2^) or effort (items individual^−1^ h^−1^) revealed that the interpretation of a polluted site varies with units, highlighting the need for consistency in the field. Litter density varied from 0.06 to 13.89 items m^−2^ and 5.2–706 items individual^−1^ h^−1^. Plastics were the major litter type constituting 75% of all litter (mean = 74.2 ± 12.5% across all sites), while clothing (10.9 ± 9.5%) and fishing gear (3.7 ± 5.2%) were also prevalent. Two items constituted > 40% of all collected litter, namely plastic beverage bottles (average 23.7 ± 17.1%, range 0–72.9%) and plastic bags (average 19.7 ± 13.2%, range 1–50.3%). Prioritizing intervention measures on these two items could reduce litter leakage significantly. Furthermore, a holistic approach was undertaken through voluntary engagement, citizen science involvement, stakeholder dissemination, and school visits to increase public awareness. Clean-up data when disseminated to relevant policymakers and stakeholders can motivate activity for the environmental management of anthropogenic litter and plastic pollution.

**Supplementary Information:**

The online version contains supplementary material available at 10.1007/s11356-025-36528-8.

## Introduction

Environmental pollution due to mismanaged anthropogenic litter (AL) is one of the most pressing issues facing the world today, and evidence of its negative socioeconomic, cultural, health, and ecological implications is well documented (Gall and Thompson 2015; Law 2017; Beaumont et al. 2019). Litter comprising different material types, such as plastics, cloth, metal, wood, ceramic, glass, rubber, and paper, is generated from different sources, including homes, tourism, fishing, shipping, and commercial sectors (Barnardo and Ribbink 2020). The majority of AL is not collected and disposed of sufficiently and accumulates in the environment (Lebreton et al. 2017). Of the AL types, plastics are considered most problematic for the environment owing to its durability, persistence, and widespread usage (Corcoran et al. 2009). Plastic litter entering water bodies annually, through rivers, storm waters, and wind, is estimated at 19–23 million tons (Borrelle et al. 2020). Plastic litter can entangle aquatic species (Gall and Thompson 2015), and both fishing and recreational boats engines potentially inflict negative economic impacts on areas that rely on fishing and tourism for their livelihood (Beaumont et al. 2019). As plastic litter moves through the environment, it is broken down into smaller pieces known as microplastics (< 5 mm). Microplastics have been documented globally across geographic regions, environmental compartments, and biological groups (Mihai et al. 2022). Microplastics are ingested by numerous species of different feeding types, including those that are important in conservation (Ryan et al. 2016), nutrition (Biginagwa et al. 2016; Sparks et al. 2021), and the economy (Calderon et al. 2019; Adika et al. 2020). Microplastics also carry toxic chemicals (Khan et al. 2021, 2022), pathogens (Rasool et al. 2021), and invasive species (Miller et al. 2018) across the areas they traverse.

To reduce plastic litter and AL in general, clean-up events have increased globally (Vincent et al. 2017; Syberg et al. 2020; Aslam et al. 2022; Catarino et al. 2023; Uogintė et al. 2024). Clean-ups, typically focusing on beaches and coastal regions (Bravo et al. 2009; Watts et al. 2017; Nelms et al. 2017), have become popular not only in reducing visible amounts of litter, but also for their efforts in community outreach, education, and the involvement of citizen scientists (Syberg et al. 2020; Catarino et al. 2023; Uogintė et al. 2024), where the term citizen science specifically refers to members of the public voluntarily participating in the scientific process through, for example, the collection of samples and/or data (Syberg et al. 2018). Additionally, clean-ups are becoming scientifically valuable in collecting data and providing real-time comparable results from previously unreported areas and on the relative importance of different types of AL (Nelms et al. 2022; Catarino et al. 2023). The International Coastal Clean-up (ICC) campaign, organized annually by the Ocean Conservancy, is among the leading global citizen science-based initiatives that operate in many countries and have demonstrated great positive impacts on the environment by stimulating regular local clean-ups. In 2023, the ICC campaign brought together 469,482 volunteers who retrieved 15,519,392 items of litter from the environment (Ocean Conservancy 2023). When such data is utilized effectively, it can be a useful tool to guide informed decisions on the management of litter, including influencing environmental policies (Jorgensen et al. 2021). Despite such worldwide success, most of the available citizen science-based published works are based in developed countries and mostly in marine environments, and only a few have focused on freshwater bodies, and very few in the African Great Lakes (AGLs) (Mayoma et al. 2019; Alimi et al. 2021).

Research on anthropogenic/plastic litter and waste management in Africa and its Great Lakes remains in its infancy compared to other regions of the world (Khan et al. 2018; Blettler et al. 2018; Alimi et al. 2021). However, microplastic research specifically in East African waters is increasing (Biginagwa et al. 2016; Egessa et al. 2020; Mayoma et al. 2020; Kosore et al. 2022; Nchimbi et al. 2022b, 2022a, 2024; KeChi-Okafor et al. 2023), and African countries have implemented mechanisms to tackle plastic litter, such as plastic bag bans in Rwanda, Kenya, and Tanzania (Carlos Bezerra et al. 2021). The first published work on the presence of AL in the African Great Lakes (AGLs) was from the Tanzanian waters of Lake Victoria and reported the presence of litter from fishing-related activities (94%), followed by plastic (4%) and cloth (2%) (Ngupula et al. 2014). The first published work on beach clean-up studies in AGLs was from a series of clean-ups between 2015 and 2018 on the shores of Lake Nyasa (Malawi) (Mayoma et al. 2019). During this 3-year clean-up campaign by the Beach and Underwater Clean-up Organization, over 2000 volunteers were able to remove a total of 490,064 items of litter, of which 80% was plastic.

**Fig. 1:**
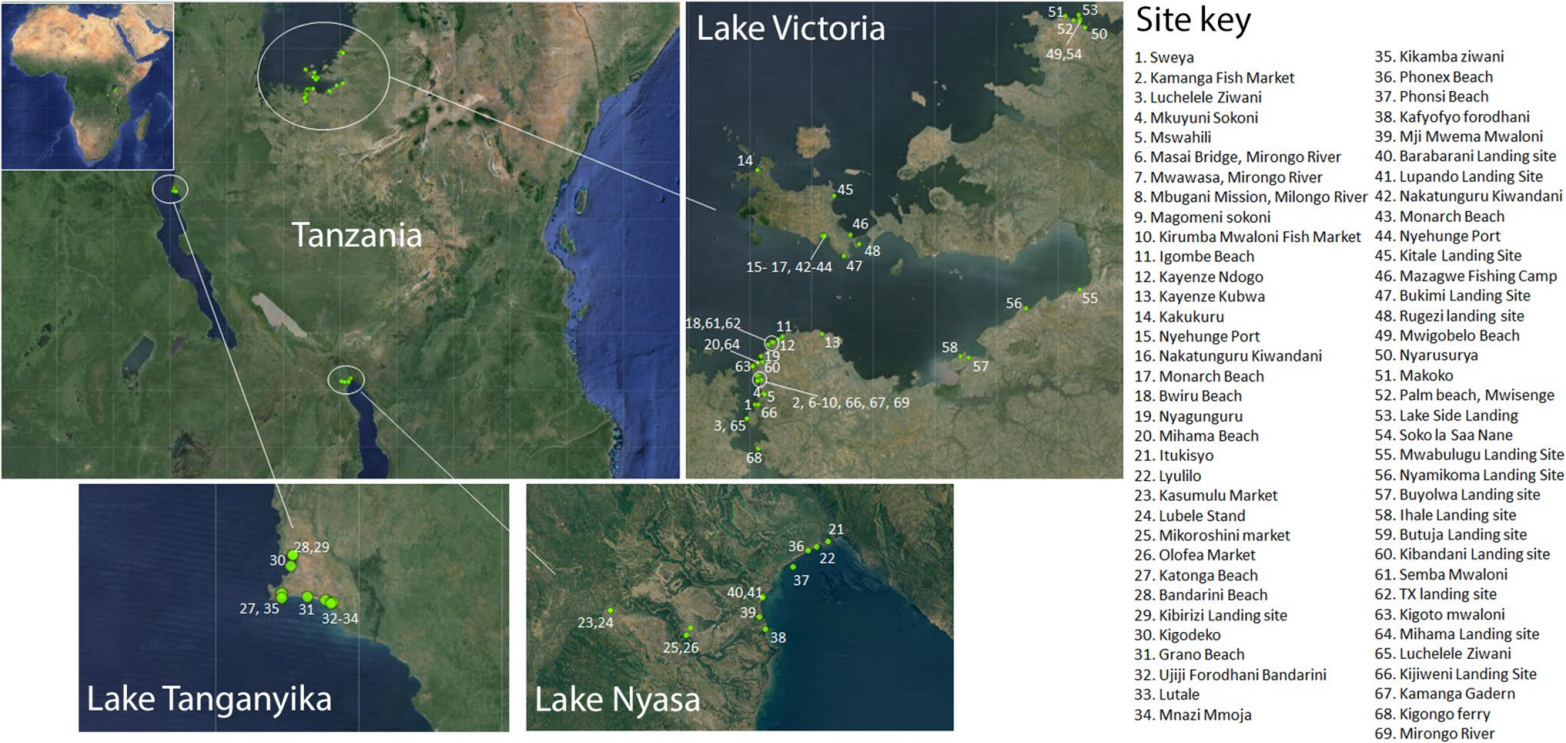
Map of clean-up site. Main map showing the locations of clean-up sites along the Tanzanian shoreline of the African Great Lake (inset: map of Africa). Numbered clean-up sites are shown on the map of each lake—Lake Victoria, Nyasa, and Tanganyika. The key to site numbers provides the name of each cleanup location

**Fig. 2:**
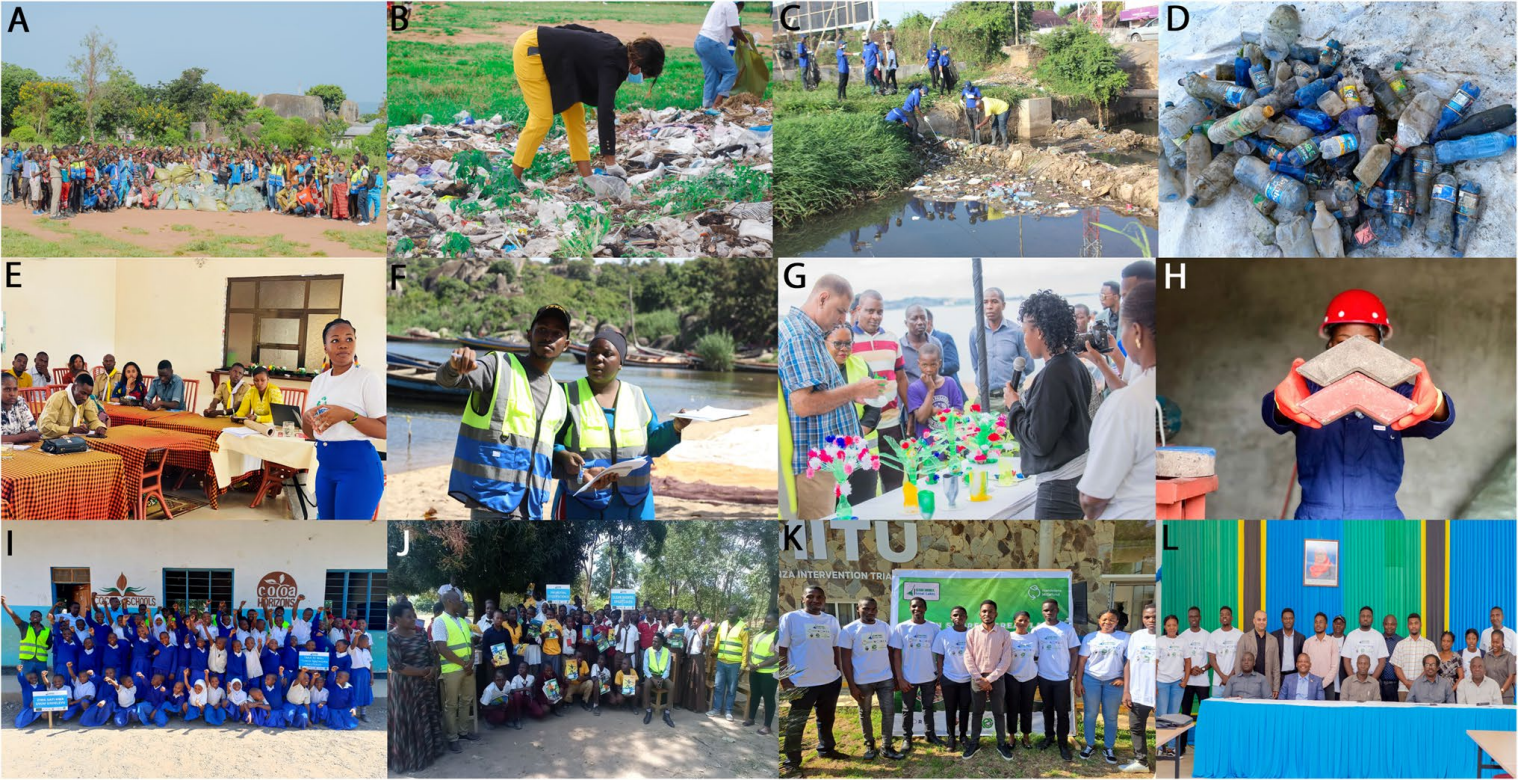
Images from the activities undertaken within the Clean Shores, Great Lakes project. Images **A**–**C** show clean-up activities at clean-up events where waste audit was performed (**D**). Training courses prior to clean-ups (**E**) were used to prepare citizen scientists (**F**). Second life products produced from recycled plastics were showcased at organized events (**G**) and eco-bricks made from collected plastic litter were used to exemplify, promote, and educate on circular economy (**H**). The project visited a number of schools engaging students at both primary and secondary levels (**I**, **J**). The end of project stakeholder meeting gathered volunteers (**K**, **L**) and diverse range of interested parties to discuss potential data-driven mitigation strategies

In the present study, we report on the results of the Clean Shores, Great Lakes project in which we conducted 69 coordinated clean-ups along the Tanzanian shorelines of the African Great Lakes, namely Lakes Victoria, Tanganyika, and Nyasa (Malawi) (Fig. 1). We further trained and mobilized local communities as environmental ambassadors through active involvement in the clean-ups, recorded clean-up data through citizen science to pinpoint hotspots and sources of litter and promoted circular economy and sustainable solutions with the goal of providing data-led advice to regional and national policymakers on mitigation strategies (Fig. 2).

## Materials and methods

### Study sites

The “Clean Shores, Great Lakes” clean-up campaign was conducted in two phases from February to April 2023 (35 sites) and August to September 2023 (34 sites) along the Tanzanian shores of the African Great Lakes (AGLs), namely Victoria, Tanganyika, and Nyasa (Fig. 1). Lake Victoria is the largest lake on the African continent and the second largest globally, covering an area of 68,800 km^2^ with a mean depth of 40 m and a maximum depth of 80 m, and is shared by Tanzania, Uganda, and Kenya. Lake Tanganyika is the second largest lake on the continent and fifth globally, covering an area of 32,900 km^2^ with a mean depth of 570 m and a maximum depth of 1470 m. It is shared by Tanzania, the Democratic Republic of the Congo, Zambia, and Burundi. Lake Nyasa, also known as Lake Malawi, is the third largest lake on the continent and the ninth largest globally, with an area of 29,600 km^2^ mean depth of 264 m, and a maximum depth of 700 m and is shared by Tanzania, Malawi, and Mozambique (Bootsma and Hecky 2003). The three lakes combined are the richest freshwater ecosystems in the world and support a large human population of 50 million-plus people with high rates of population growth (Ogutu-Ohwayo and Balirwa 2006). The AGLs connect inland East Africa to the open oceans and Europe through the Mediterranean Sea, the Atlantic, and Indian Oceans via the Nile, Congo, and Zambezi Rivers, respectively. The Tanzanian side of the AGLs is dominated by fishing, transportation, agriculture, mining, tourism, and trade-related activities that have contributed to the growing number of urban settlements, including the Tanzanian second-largest city of Mwanza.

An overview of the 69 clean-up sites is provided in Supplemental Table S1 which details each site (location, date visited, district, administrative area, lake basin, and primary activities), the clean-up (area covered, time taken, number of volunteers), waste audit (% of waste audited, total weight and total items, as well as items normalized to area and individuals per hour), and the enumeration of items within each waste category (plastic, cloth, paper, fishing gear, glass, sanitary, metal, medical, electrical, rubber, ceramic, and wood). A summary of the clean-ups is provided in Table 1 with the 69 sites divided by administrative area. In Lake Victoria, clean-ups were conducted on the eastern part of the lake and were conducted in four administrative areas with the majority (27 sites) conducted in largest area by size and population density, Mwanza City. Ukerewe District (11), Musoma Municipality (6), and Busega district (4) were the other administrative areas visited at Lake Victoria. In Lake Tanganyika, the focus was on Kigoma Municipality (9), which is a highly urbanized area in Kigoma Region, and in Lake Nyasa, clean-ups were conducted in Kyela District (12) (Table 1). Across administrative areas fishing-related activities were dominant, along with trading (markets), transportation (ports), and tourist sites (Table 1, Supplemental Table S1).

**Table 1:** Summary of the clean-up sites according to administrative area. The site numbers can be cross referenced for identification with the key in Fig. 1. The primary activities within each set of sites are provided along with the number of volunteers participating within each administrative area. The area covered, litter items collected, and total weight of litter collected within the administrative area are also shown. The full dataset detailing the site information, clean-up specifics, and the amount and categorization of the collected litter is found in Supplemental Table S1

Administrative area	Great Lake	Population size^a^	Number of sites	Sites numbers	Primary activities	Volunteers	Area covered (m^2^)	Litter items collected	Litter weight (kg)
Mwanza city	Victoria	594,834	27	1–13, 18–20, 59–69	Fishing camp and landing site, tourism, transportation, trade,	2377	324,988	206,433	16,047
Ukerewe District	Victoria	387,815	11	14–17, 42–48	Fishing camp and landing site, tourism, transportation, settlement	769	50,887	53,736	2885
Musoma Municipality	Victoria	164,172	6	49–54	Transportation, fish landing site, trade	302	61,139	12,808	614
Busega District	Victoria	282,167	4	55–58	Fish landing site	121	44,565	13,606	727
Kigoma Municipality	Tanganyika	232,388	9	27–35	Fishing camp, tourism, transportation, settlement, fish landing site	652	131,424	81,850	2596
Kyela District	Nyasa	266,426	12	21–26, 36–41	Fishing, tourism, transportation, trade, agriculture	1262	150,242	62,895	3112
Total			69			5483	763,245	431,328	25,981

### Clean-up methodology

The clean-up campaign was carried out according to standard protocols developed by the African Marine Litter Monitoring Manual (Barnardo and Ribbink 2020), with some modifications from the International Coastal Clean-up Protocol (Ocean Conservancy 2023) and the Danish mass experiment community clean-up protocol (Syberg et al. 2020) to align with freshwater environments. We selected the sites based on accessibility (not blocked by private owners or jetties) and land use characteristics, such as settlement, fishing, tourism, trade, and transportation (Supplemental Table S1). Urban and remote areas representing different natural and man-made features, such as bays, gulfs, river mouths, and drainage ditches, were covered. We have divided the methodology into pre-clean-up, clean-up, and post-clean-up.

### Pre-clean-up and outreach program

Before the start of clean-up events, volunteers from various local community groups and organizations that are directly or indirectly involved in litter collection were selected and recruited for training as part of the project’s capacity-building. A total of 25 volunteers were recruited and underwent 3-day training on clean-up best practices that would later make them trainers during the clean-up campaign (i.e., “Train the Trainer” model (Cheung et al. 2018)). Centrally training of volunteers, comprising of university students, waste pickers, scouts, and NGOs, ensured reproducible data collection during clean-ups. This was followed by the launching event held in Mwanza City on February 27, 2023. The event was attended by over 300 participants from various stakeholder groups, including government officials, fishers’ organizations, jogging clubs, individual activists, and civil society organizations, who together received widespread media coverage. The deputy mayor of Mwanza City delivered a keynote speech to the participants. Local leadership was consulted for site selection, community mobilization, and access to sites before clean-up day.

### Clean-up activities

In collaboration with the local community, we identified sites such as markets, rivers, and beaches that cause litter influx to the AGLs. Clean-ups were designed to minimize interference with the daily routine of the community while at the same time increasing volunteer participation. This was done by ensuring that clean-ups were scheduled in the morning hours most often from around 7 a.m. and lasting for 1 to 2 h. To avoid damage to ecosystems, we did not use mechanical equipment as our clean-up tools and techniques were limited to hand picking (Griffin et al. 2018). Only macro-litter (item > 2 cm) visible at the surface of the sand or shallow water was collected by hand.

Upon arrival at clean-up sites, the length and width of the clean-up sites were recorded. Each volunteer was registered and provided with equipment such as collection bags, gloves, and masks. Volunteers were advised to wear gumboots or closed shoes to minimize the risk of injuries from sharp objects. At each site, location, site name, area covered, and start and end time were recorded (Barnardo and Ribbink 2020; Ocean Conservancy 2023). Additionally, the number, age, and gender of the volunteers were also recorded in data collection sheets (Syberg et al. 2020). The start and end time of the clean-up event was also noted. All site data including the size of the site, duration of the clean-up, and volunteer composition is available in Supplemental Table S1.

### Post-clean-up processing and waste management

Post-clean-up protocols were conducted according to previously established methodologies (Barnardo and Ribbink 2020; Ocean Conservancy 2023). Briefly, litter collected in bags were aggregated and weighed (in kg) before undergoing waste audit. A representative sample of at least 10% of total litter collected was taken randomly for waste audit (average = 21.4 ± 10.4%, range = 10.0–66.6%, Supplemental Table S1). AL was classified into 12 main categories with each category containing a number of individual sub-types: plastics (30 types), fishing gear (4), rubber (7), sanitary products (7), medical products (5), clothing (4), paper and cardboard (8), wood (5), metals (8), glass (4), pottery and ceramics (2), and electrical waste (2). As main category of litter, plastics were grouped as being related to: cigarettes (e.g., lighters, wrapping), food (e.g., beverage bottles, food containers, cups and plates, food wrappers), plastic bags, hard plastics (e.g., buckets, jerry cans), soft plastics (industrial sheeting, mosquito nets), and other plastics (e.g., polystyrene/foam pieces and packaging). All litter items are available in the Supplemental Table S1.

Post-waste audit, further classification was made to group litter as recyclable and non-recyclable, after which the recycled litter was handed over to the local recyclers, whereas non-recyclable litter was transported to the nearby designated landfills for disposal.

### Data handling

We recorded data using both predesigned hardcopy and soft copy forms as previously described (Barnardo and Ribbink 2020). The soft copy forms were filled out through Kobo Collect App version 2023.2.4, an online data collection tool. We used the app to minimize risks of data loss and ensure effective data collection and compilation. Hard copy forms were used as backups in case of faults or errors in the App. Enumerated litter data obtained from percentages of total litter assessed was multiplied-up to account for 100% of the litter, with the assumption made that the random selection of assessed litter was representative of the whole. The compiled dataset of each clean-up site, together with photos of the event, was uploaded to the project website https://cleanshoresgreatlakes.norceresearch.no/.

### Data presentation

**Fig. 3:**
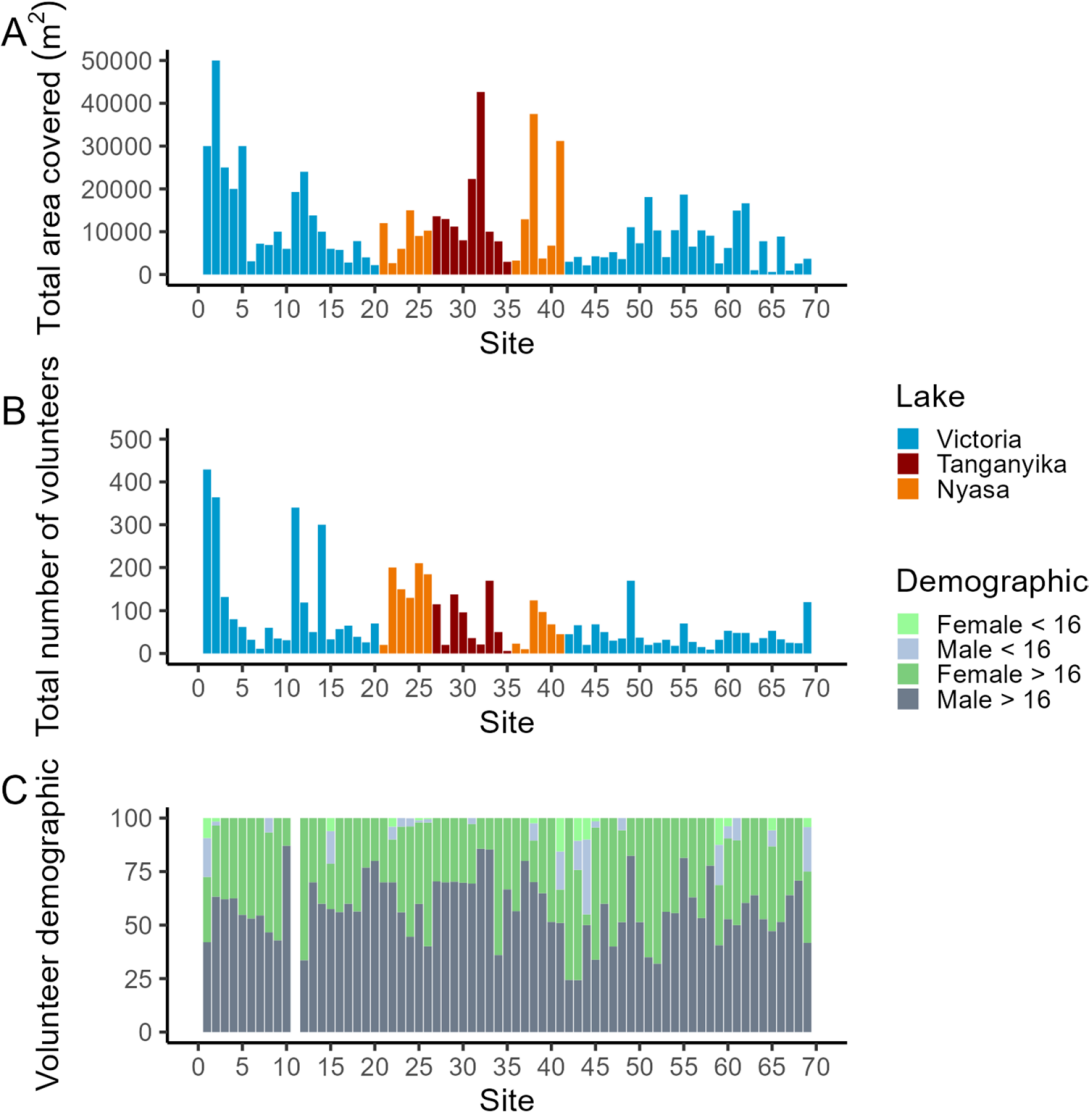
Area covered (**A**) and volunteer numbers (**B**) at each of the 69 clean-up sites. The distribution of volunteers between categories of adult males, adult females, child males (< 16 years old), and child females (< 16 years old) is shown (**C**). Volunteer demographic data was not collected at site number 11 (Igombe Beach)

**Fig. 4:**
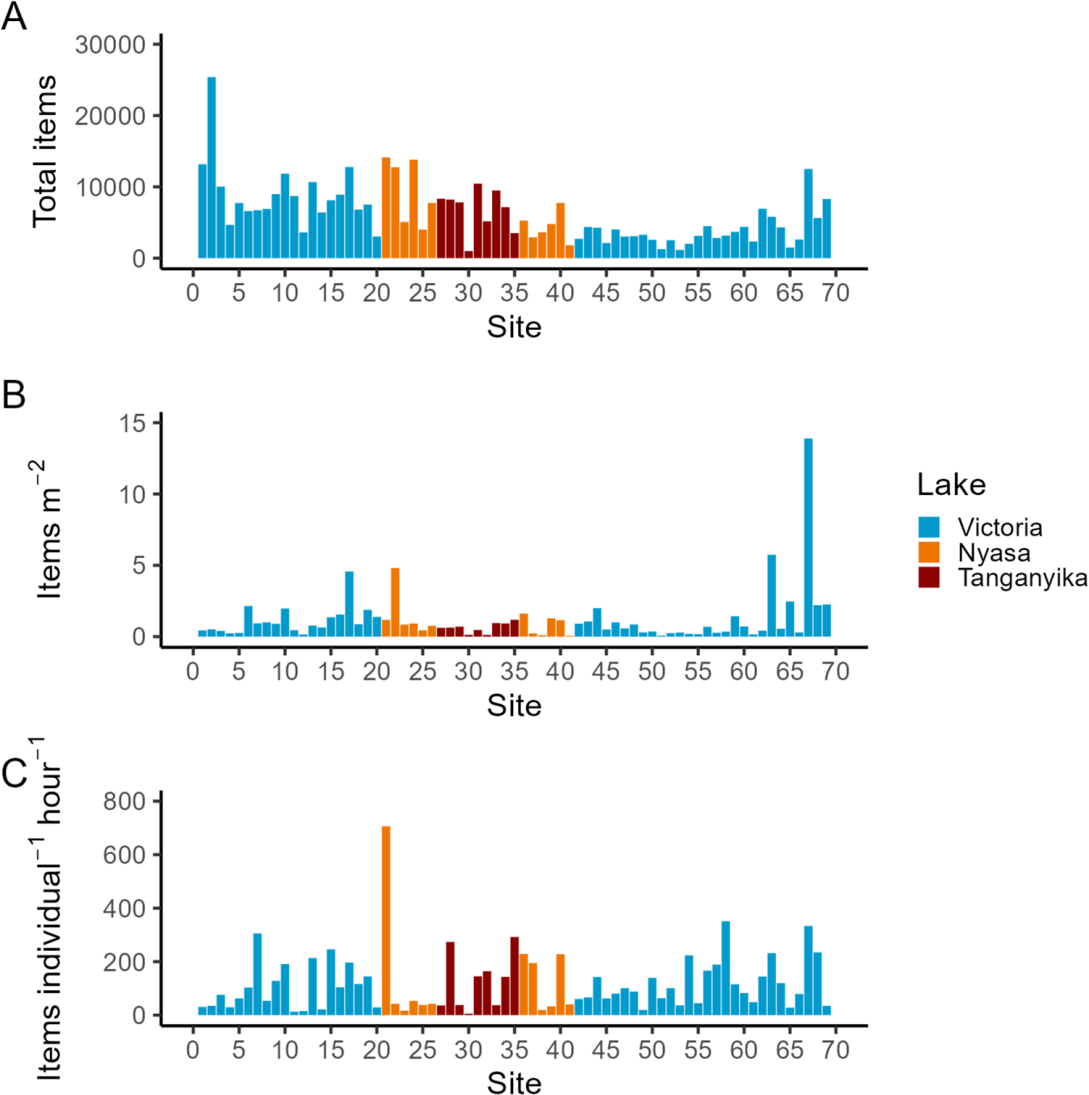
Litter density across the 69 clean-up sites shown as total items collected (**A**), and then normalized to area (items m^−2^, **B**) and to the number of volunteers and duration of the clean-up (items individual^−1^ h.^−1^, **C**)

**Fig. 5:**
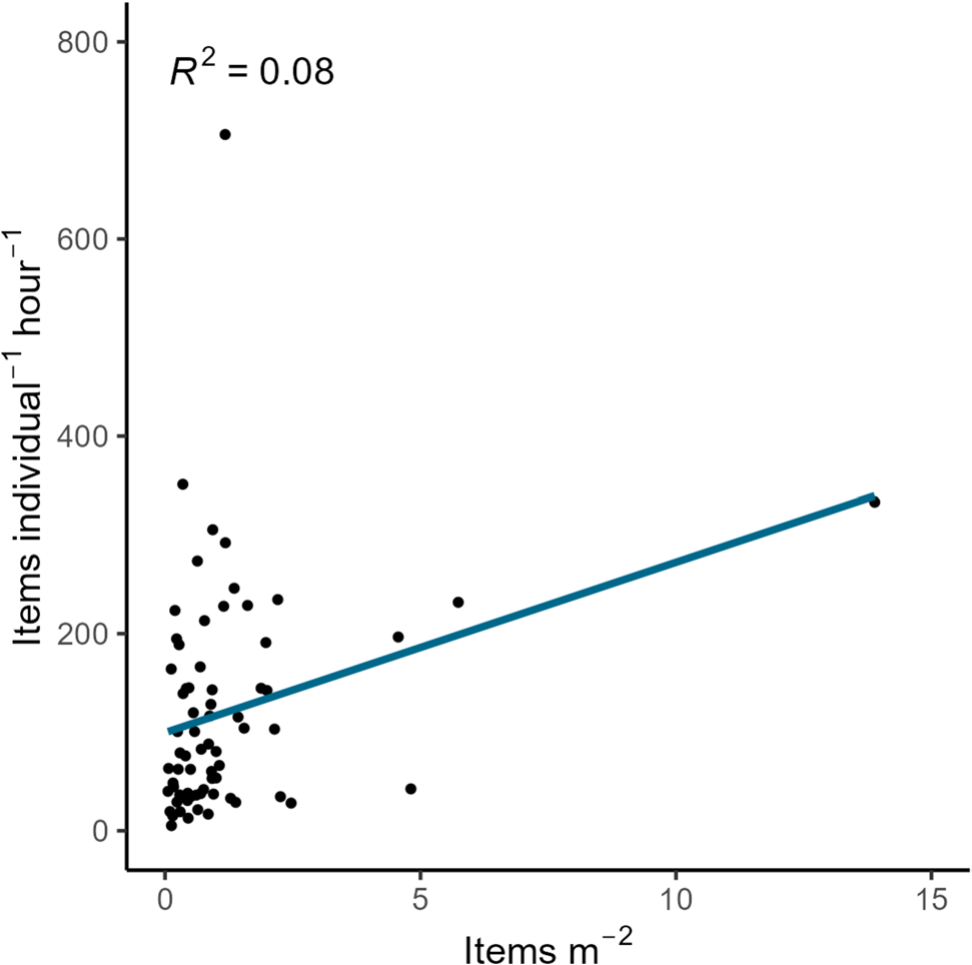
The correlation between the collected litter at each site (*n* = 69) when expressed per area (items m^−2^) or normalized to the number of volunteers and duration of the clean-up (items individual^−1^ h^−1^) shows no obvious linear relationship

**Fig. 6:**
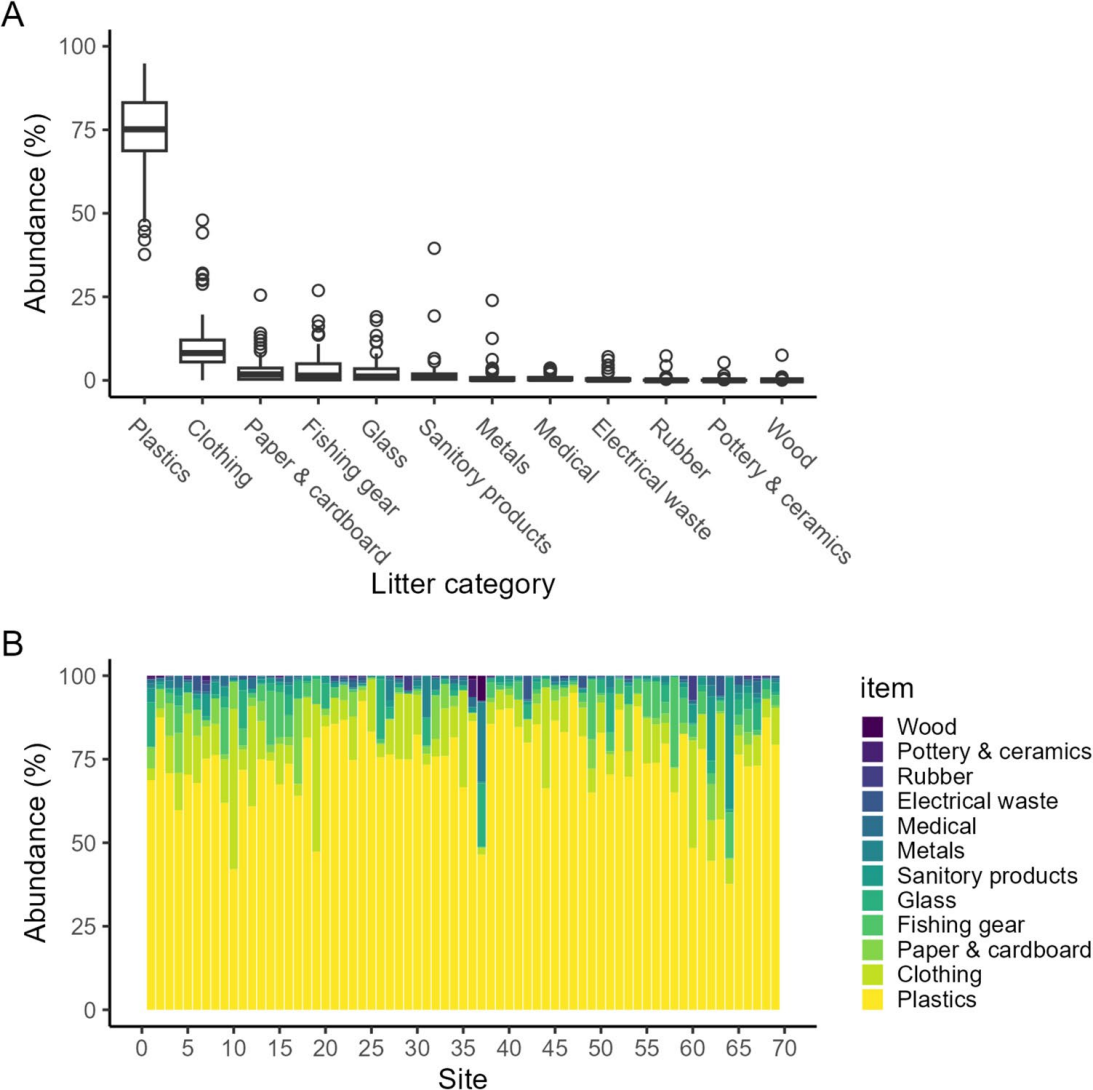
Abundance of litter categories (in %) from clean-ups (*n* = 69). **A** Boxplots display first quartile, median, third quartile, and 1.5 * interquartile length (lower and upper whiskers). Observations above or below the whiskers are considered outliers and are shown with open circles. **B** The distribution of the litter categories across the 69 clean-ups is shown

**Fig. 7:**
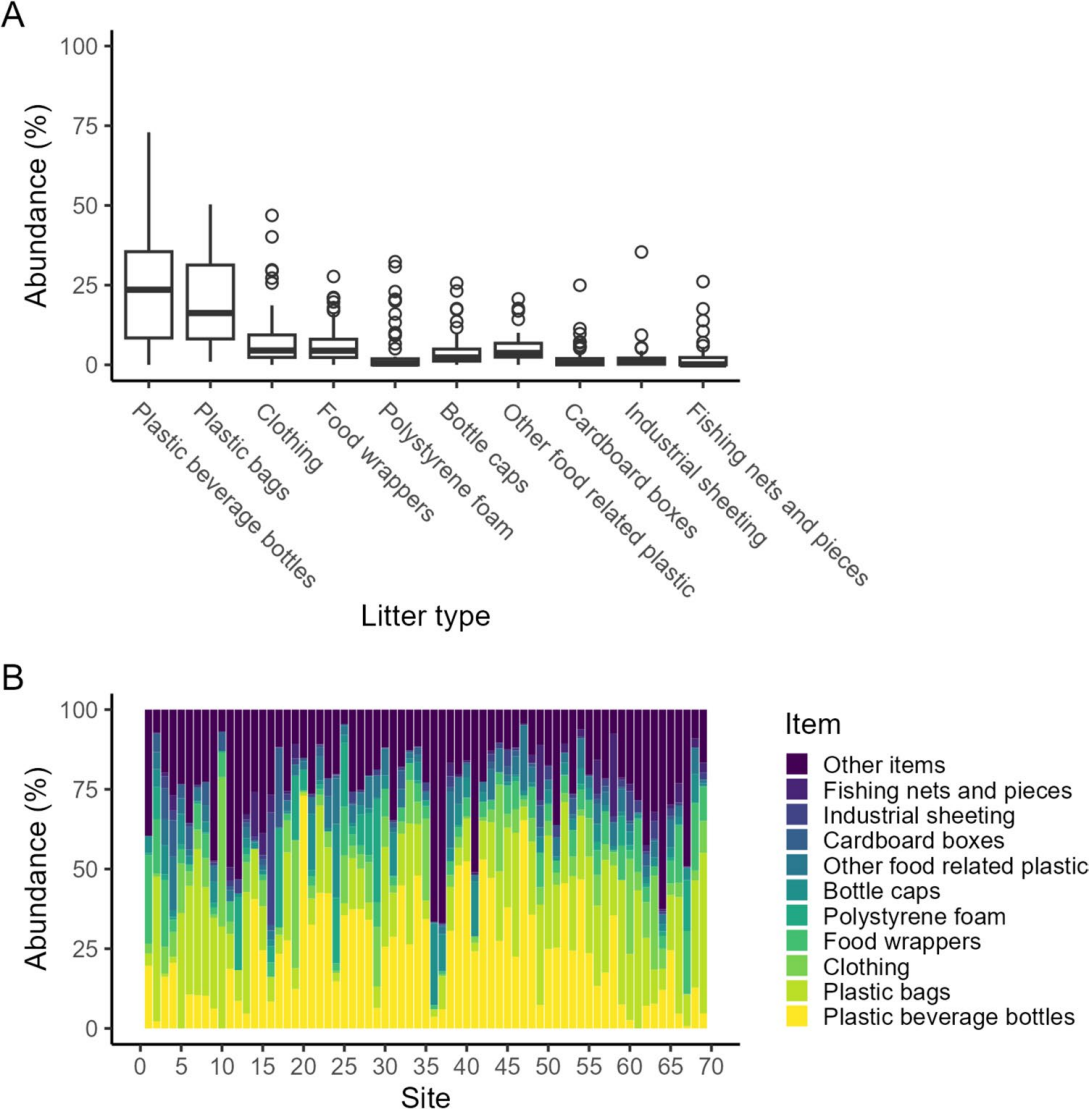
Abundance of the top 10 items (in %) from clean-ups (*n* = 69). **A** Boxplots display first quartile, median, third quartile, and 1.5 * interquartile length (lower and upper whiskers). Observations above or below the whiskers are considered outliers and are shown with open circles. **B** The distribution of the top 10 items (and “other items” category) across the 69 clean-ups is shown

Data was tabulated in Microsoft Excel (Supplementary Table S1). Mean values ± standard deviation is stated unless otherwise stated. Figures 3, 4, 5, 6, and 7 were generated in R studio (2023.12.1 Build 402), using tidyverse and the ggplot2 package.

## Results and discussion

Overall, Clean Shores, Great Lakes organized 69 clean-ups with 48 sites around Lake Victoria, 12 sites around Lake Nyasa (Malawi), and nine sites around Lake Tanganyika. A total of 5483 volunteers engaged with the project across all clean-ups. In total, 431,328 pieces of anthropogenic litter were collected weighing 25,981 kg. With respect to outreach activities, of 21 schools (10 secondary and 11 primary) were visited, and over 3000 students were directly engaged with the project’s awareness campaign. The stakeholder meeting held at the end of the second campaign was attended by a diverse group of interested parties. The results of the study are discussed in the following sections by site, by litter type, and then with a focus on the plastic waste. Following this is a precis of the outreach, engagement, and recommendations offered by the project.

### Variation in site area and volunteer composition

Across the 69 clean-up sites, there was great variation in the size of the areas cleaned from 603 m^2^ at Luchelele Ziwani (Lake Victoria, site 65) to 50,000 m^2^ at Kamanga Fish Market (Lake Victoria, site 2) (Fig. 3A). Similarly, there was great variation in volunteer numbers from 429 people attending the first clean-up event at Sweya (Lake Victoria, site 1) to only six volunteers at Kikamba Ziwani (site 35, Lake Nyasa) (Fig. 3B). Overall, the largest group of participants by gender and age was males > 16 years old (58 ± 15%) followed by females > 16 years old (38 ± 15%), male < 16 years old (3 ± 7%), and female < 16 years old (1 ± 3%). While men were usually the largest volunteer group at each site (Fig. 3C), there were a few exceptions where women volunteers were greater, notably at sites 12 (Kayenze Ndogo, Lake Victoria), 33 (Lutale, Lake Tanganyika), and 42 (Nakatunguru Kiwandani, Lake Victoria). Children (i.e., males and females < 16 years old) were best represented at site 44 (Nyehunge Port, Lake Victoria) where they made up 45% of the volunteers.

### Variation in AL across the different sites

Litter can be expressed as the weight or items collected (Hengstmann and Fischer 2020), but the majority of scientific literature on the topic has chosen the metric of items (Watts et al. 2017; Mayoma et al. 2019; Egessa et al. 2020; Syberg et al. 2020). Total items ranged from 1003 at site 30 (Kigodeko (fish landing site), Lake Tanganyika) to 25,380 items at site 2 (Kamanga Fish Market, Lake Victoria) (Fig. 4A). However, absolute measures are of little value and clean-up data is typically presented as items of litter collected normalized to the metric of the size of the area covered (i.e., items per unit area (Watts et al. 2017; Mayoma et al. 2019; Egessa et al. 2020; Syberg et al. 2020) or the amount of effort used in terms of time and number of people involved (Nelms et al. 2017)). Since there is no clear agreement on which unit is better and that both site area and volunteer numbers exhibit great variation in our study (Fig. 3), we present our site data in both forms.

Litter abundance when normalized to area varied from 0.06 to 13.89 items m^−2^ across all sites. Kamanga Garden (site 67 (tourism and fish market), Lake Victoria) recorded the highest litter density 13.89 items m^−2^ followed by site 63 (5.74 items m^−2^, Kigoto mwaloni (fish landing site), Lake Victoria), site 22 (4.81 items m^−2^, Lyulilo (trade), Lake Nyasa), and site 17 (4.56 items m^−2^, Monarch Beach (tourism and market), Lake Victoria). Litter densities at the other sites were below 2.5 items m^−2^ with the lowest recorded density of 0.06 items m^−2^ at site 41 (Lupando Landing Site (fishing camp), Lake Nyasa) (Fig. 4B). Our findings are higher than those reported in most North American Great Lakes (Hoellein et al. 2015), Lake Tollense in Germany (Hengstmann and Fischer 2020), and Lake Nyasa (Mayoma et al. 2019).

When normalized to the amount of litter collected per volunteer per time spent metric, the abundance ranged from 706 items individual^−1^ h^−1^ at site 21 (Itukisyo (tourism), Lake Nyasa) to 5.2 items individual^−1^ h^−1^ at site 30 (Kigodeko (fish landing site), Lake Tanganyika) (Fig. 4C). The amount of litter collected expressed by this metric at Itukisyo was twice as much as at the next sites, site 58 (351 items individual^−1^ h^−1^, Ihale Landing site (fish landing site), Lake Victoria), site 67 (333 items individual^−1^ h^−1^, Kamanga Garden (tourism), Lake Victoria), and site 7 (305 items individual^−1^ h^−1^, Mwawasa on the Mirongo River (trade and transportation), Lake Victoria). Our findings are generally lower, but in the range of Nelms et al. (2017) who reported a range of 51–1978 items individual^−1^ h^−1^ on British beaches. However, litter items on British beaches may originate from both marine- and land-based activities; direct comparisons to the African Great Lakes cannot be made.

Overall, there was no linear relationship between litter densities when expressed by the two metrics (*R*^2^ = 0.08, Fig. 5), suggesting that how data is normalized affects the interpretation of relative pollution between sites (Hengstmann and Fischer 2020). The top 10 sites with the most abundant litter under two metrics show a broad disparity (Table 2) with only three sites, namely Kamanga Garden (site 67, tourism), Kigoto mwaloni (63, fish landing site), and Kigongo ferry (68, transportation) appearing on the top 10 list under both metrics. Kamanga Garden which has the highest abundance overall with 13.89 items m^−2^ ranks fourth under individual^−1^ h^−1^. Regardless of the disparity, sites where the main activity is related to fishing (market, landing site), transportation (ferry ports), trade (general markets), and/or tourism are well represented on both lists. Fishing activities have been reported to cause potential leakage of litter (Nguyen et al. 2022). Similarly, recreational activities have been reported as main source of AL in Zanzibar (Maione 2021). However, since many sites have more than one activity, using activity as a causation for litter density is difficult. This, in turn, makes suggestions for targeted mitigation challenging (discussed further in the “Data-driven recommendations” section).

**Table 2:** Top 10 sites for anthropogenic litter according to two different expression metrics: Items m^−2^ and Items individual^−1^ h^−1^

Litter density expressed as Items m^−2^					Litter density expressed as Items individual^−1^ h^−1^		
Rank	Site name (number)	Main activities	Items m^−2^		Site name (number)	Main activities	Items individual^−1^ h^−1^
1	Kamanga Gadern (67)	Tourism, Market (fish)	13.9	1	Itukisyo (21)	Tourism, Settlement	706
2	Kigoto mwaloni (63)	Fish landing site	5.7	2	Ihale Landing site (58)	Fish landing site	351
3	Lyulilo (22)	Trade, Settlement	4.8	3	Kamanga Gadern (67)	Tourism, Fish market	333
4	Monarch Beach (17)	Tourism, Market	4.6	4	Mwawasa, Mirongo River (7)	Trade, Transportation	305
5	Luchelele Ziwani (65)	Fish landing site, Settlement	2.5	5	Kikamba ziwani (35)	Fish landing site, Settlement	292
6	Mirongo River (69)	Trade, Transportation	2.3	6	Bandarini Beach (28)	Transportation, Trade	274
7	Kigongo ferry (68)	Transportation, Landing site	2.2	7	Nyehunge Port (15)	Transportation, Trade	246
8	Masai Bridge, Mirongo River (6)	Trade, Settlement	2.1	8	Kigongo ferry (68)	Transportation, Fish landing site	234
9	Nyehunge Port (44)	Transportation, Settlement	2.0	9	Kigoto mwaloni (63)	Fish landing site,	231
10	Kirumba Mwaloni Fish Market (10)	Market (fish), Ferry transportation	2.0	10	Phonex Beach (36)	Tourism. Trade	228

### Abundant litter categories

Plastic made up 75% of all litter collected (mean = 74.2 ± 12.5% across all sites), while clothing (10.9 ± 9.5%), fishing gear (3.7 ± 5.2%), paper (3.3 ± 4.4%), glass (2.8 ± 4.0%), and sanitary products (2.1 ± 5.2%) were the next most prevalent categories. Other litter categories constituted less than 2% each, and wood was the least prevalent category overall with a 0.1% share (Fig. 6A). The composition of litter category and distribution varied across sites: plastic (37.5–94.9%), clothing (0–48%), fishing gear (0–26.9%), paper (0–25%), glass (0–19%), sanitary (0–39.7%), metal (0–23.9%), medical (0–3.6%), electrical (0–8%), rubber (0–7.3%), ceramic (0–5.3%), and wood (0–7.5%) (Fig. 6B).

Our findings agree with other published works locally and globally. In Lake Nyasa (Malawi), plastic litter comprised 80.2 ± 5.0% of all AL (Mayoma et al. 2019) and in Lake Michigan, plastic represented 75% of total litter (Hoellein et al. 2015). The categorization of litter in the present study is based on established protocols (Barnardo and Ribbink 2020). The broad category of “plastics” encompassing a wide variety of products and sources (cigarettes, food (including bottles, wrappers, and cutlery)), plastic bags, hard plastics, soft plastics, and packaging materials, after which clothing and fishing gear were most prevalent. These two categories have themselves become regarded as a portion of the plastic waste. Textiles have been shown to be a source of microfibers (Athey and Erdle 2022) which have been found within the environment, including in the marine wasters of the East African coast, although not all fibers are synthetic in origin (KeChi-Okafor et al. 2023). Similarly, abandoned, discarded, or otherwise lost fishing gear (ADLFG) have been reported in Lake Victoria (Egessa et al. 2020), North America Great Lakes (Vincent et al. 2017), and Vietnamese waters (Nguyen et al. 2022). Defining “plastics” to also include these categories would increase the proportion plastic waste found during all the clean-ups to close to 90%.

### Relative abundance of litter items

Of the 431,328 items collected during the clean-ups, plastic beverage bottles (89,766 items, 20.8%) and plastics bags (85,605 items, 19.8%) were the dominant items each accounting for about 20% of all litter and combined greater than 40%. Both items were almost ubiquitously present at all clean-up locations, although contributions differed (Fig. 7). Plastic bottles (average 23.7 ± 17.1%, range 0–72.9%) were not found at only one site, Semba Mwaloni (site 61, Lake Victoria) a fishing camp. Plastic bags (average 19.7 ± 13.2%, range 1–50.3%) were found during all clean-ups.

We adopted the Ocean Conservancy (2023) protocol by identifying the top 10 items that are found across clean-up sites. The top 10 items accounted for 76% of all litter, seven of which were within the plastic litter category, two of which were clothing and fishing nets and pieces that be considered plastics under different taxonomies, and cardboard boxes as the only non-plastic-related item: (1) plastic bottles (20.8%), (2) plastic bags (19.8%), (3) cloth/towel (7.9%, range across site 0–46.9%), (4) food wrappers (6.8%, range 0–5.8%), (5) polystyrene/foam (5.2%, range 0–32.5%), (6) plastic bottle caps (4.7%, range 0–26.7%), (7) other food-related plastics (4.6%, range 0–20.7%), (8) cardboard boxes (2.7%, range 0–25.0%), (9) industrial sheeting (1.9%, range 0–35.4%), and fishing nets and pieces (1.6%, range 0–26.%) (Fig. 7). The remaining litter items were each recorded below 1.5%. The types of litter found in the African Great Lakes are in general comparable with studies reported from elsewhere (Ngupula et al. 2014; Mayoma et al. 2019; Nguyen et al. 2022; Ocean Conservancy 2023). Ocean Conservancy (2023) ranked carrier bags, beverage bottles, bottle caps, and food wrappers among the top 10 plastic litter items. Similarly, our findings are in line with the ICC report for some plastic litter items: food wrappers, beverage bottles, bottle caps, and plastic bags that were listed among the top ten most collected items. The overall picture that emerges from clean-up studies globally is that the litter problem is a plastic problem.

### Recommendations and guidance

The primary objective of the Clean Shores Great Lakes project was to conduct clean-ups along the Tanzanian shorelines of the African Great Lakes. However, it is now recognized that clean-ups although effective are only one part of a holistic solution (Vincent et al. 2017; Syberg et al. 2018; Uogintė et al. 2024). Thus, within this project, a broader range of activities were undertaken to engage communities through education and outreach, disseminate our data via a stakeholder meeting, and recommend data-driven mitigation strategies (Fig. 2). We outline these efforts in the following sections.

### Education, outreach, and citizen science involvement

Twenty-one schools (10 secondary and 11 primary) in the Kyela district (Lake Nyasa) were visited at the start of second campaign in the region (August 2023). Over 3000 students were directly engaged during the project’s awareness campaign, where we held discussions with environmental clubs and showcased recycled products to students. Students were taught about the impacts of mismanaged litter and how adopting waste management hierarchies such as refuse, reduce, reuse, and recycle (4Rs) is key toward curbing the problem of plastic pollution. In some instances, students were also able to showcase various activities, such as gardening using empty cement bags, in line with the circular economy concept. Following discussions at the schools and the exhibition of recycled products, there was eagerness among students and teachers to develop a zero-waste plan that suited their schools’ needs. Students showed interest and registered to participate in future training regarding circular economy strategies that targeted plastic litter reduction. As described in the “Variation in site area and volunteer composition” section, children under the age of 16 were not well represented during clean-ups, accounting for an average of 4%. Following the school visits, children were 7.3% of volunteers in the Lake Nyasa clean-ups, but a critical assessment of the project could be that more needs to be done to engage significant numbers of younger participants. Projects using citizen scientists primarily sourced from school-aged children have reportedly shown increases in environmental awareness and perception among participants (Syberg et al. 2018, 2020; Catarino et al. 2023). The value of citizen science in clean-ups goes beyond the collection of litter. The economic benefit of volunteered hours allows for an extremely cost-effective action and involvement in clean-ups leads may lead positive changes in awareness and behavior (Nelms et al. 2017). Furthermore, data collected from citizen science data can potentially contribute to the measurement of United Nations Sustainable Development Goals targets and indicators (Fritz et al. 2019). In the present study, citizen scientists were actively involved in the collection, categorizing and recording of litter which, based on informal conversations during clean-ups, has led to a change in how participants view the issue of plastic pollution.

### Stakeholder engagement and end of project dissemination workshop

Throughout Clean Shores, Great Lakes, we conducted regular stakeholder meetings with various target groups depending on the location, including students, environmental clubs, market leaders, fishermen, local councilors, recyclers, district management, NGOs, government agencies, ministries, and the media. These engagements allowed the work of the project to spread and led to interviews and articles in the Tanzanian media. At the end of the second campaign, we gathered a diverse group of interested parties to the project’s Dissemination Workshop, including the following: Government Departments (e.g., Ministry of Fisheries and the Office of the Environment), Lake Victoria Region Local Authorities Cooperation (LVRAC) an umbrella organization for municipal policy makers, mayoral representatives of Mwanza City and the neighboring districts, academics, affected communities, e.g., fisheries workers and union representative, plastic industry representatives, organizations that partnered with us during clean-ups such as the scouts, and local and national media—the meeting was featured across the Tanzanian media. Participants viewed litter as an urgent problem that requires immediate action and attributed it to behavior and inadequate infrastructure to prevent leakage at source. Some stakeholders pointed out that the long distance between points of collection and disposal sites in some rural places and remote islands has discouraged the community from preventing littering. Inadequate law enforcement and irresponsible product manufacturers were seen as obstacles that needed to be overcome to bring about positive changes.

### Data-driven recommendations

Based on clean-up data collected in the project, two approaches could be advocated for mitigation (1) where to clean and (2) what to clean. The two normalized units can both guide informed decision-making, but both provide differing interpretations (Hengstmann and Fischer 2020). Items m^−2^ have been widely adopted for citizen science-led clean-ups and monitoring programs worldwide (Bravo et al. 2009; Nelms et al. 2017; Nguyen et al. 2022) and increasingly reported in the region (Mayoma et al. 2019; Ogello et al. 2024). In terms of where to target sites for mitigation, the area-based metric can reveal their dynamics of AL in the environment and the sources from which they originate (Browne et al. 2015), and models can incorporate such data to predict trends under various management options (Turrell 2020), but negates important factors in the success of a clean-up. Thus, the unit of litter individual^−1^ h^−1^ is also useful as number of volunteers and time taken directly influences the amount of litter collected (Araújo et al. 2018). The common denominator of the most polluted sites listed in Table 2 is rather obviously that they are where people gather—fishing sites, markets, transportation. Many the most common items found were related to food and beverages (Fig. 7). Thus, the presence of such litter is likely related to improper and inefficient waste collection and infrastructure, including limited recycling capability, which is absent from much of Africa (Adebiyi-Abiola et al. 2019). The primary recommendation for the administrative areas would be improved waste management.

The two most abundant items of litter were plastic bottles and bags. Mitigating these two items would address 40% of all collected litter. Tanzania first tried to approve a plastic bag ban in 2006, but at attempt lacked implementation. A levy was introduced in May 2015 and a total ban that prohibited the import, export, manufacturing, selling, storing, supplying, and use of plastic bags came to effect on June 1, 2019 (Carlos Bezerra et al. 2021). This promulgation led the start of a black market of single-use plastic bags (Carlos Bezerra et al. 2021) and there is likely a lag-phase before the effectiveness of the ban is reflected in litter composition. Other studies conclude that plastic bag bans serve as an instrumental tool to reduce their leakage into the environment (Martinho et al. 2017; Nielsen et al. 2019). Plastic beverage bottles are used when tap water is considered unsafe for human consumption. However, the inclusion of plastic bottles in mandatory sustainable intervention measures, such as extended producer responsibility (EPR), polluter pay principles, and deposit return schemes, may stimulate both producer- and consumer-led solutions that can bring about changes in litter release. To reduce bottle and bag litter, regulations and producer-led implementations need to be coupled to multi-facet approaches that involve the community via both participatory action and behavior changes in the population through awareness campaigns, including regular clean-ups.

## Conclusions

The data gathered within the Clean Shores, Great lakes project provides an extensive description of the nature and distribution of anthropogenic litter (mainly plastics), along Tanzanian shorelines of the African Great Lakes. Here we note the broad connection between litter density and public gathering places, where the increased availability of litter collection infrastructure would potentially lessen environmental leakage. Highlighting the two main contributing items (plastic bottles and bags) brings attention to existing single use bag bans and the prospect of extended producer responsibility actions. While the main project objective was to conduct clean-ups, the holistic approach advocated voluntary engagement, citizen science involvement, stakeholder dissemination, and school visits increases public awareness and the notion of environmental stewardship. Clean-up data when disseminated to relevant policymakers and stakeholders can motivate activity within environmental management of anthropogenic litter and plastic pollution.

## Supplementary Information

Below is the link to the electronic supplementary material.

Supplementary file1 (XLSX 61 KB)

## Data Availability

All data generated or analyzed during this study are included in this published article and its supplementary information file (Table S1. Full dataset of clean-up activities from the Clean Shores, Great Lakes project).
